# QStrain: an interactive platform for viral genome analysis and nucleic acid therapeutic design

**DOI:** 10.1186/s12859-026-06424-0

**Published:** 2026-04-10

**Authors:** Euna Jeong, Seungyean Lee, Sumin Jeong, Hansaem Lee, Kyung-Chang Kim, Joo-Yeon Lee, Minwook Shin, Sukjoon Yoon

**Affiliations:** 1 Research Institute of Women’s Health, Sookmyung Women’s University, Seoul, 04310 Republic of Korea; 2 Department of Biological Sciences, Sookmyung Women’s University, Seoul, 04310 Republic of Korea; 3 Division of Emerging Virus and Vector Research, Korea National Institute of Infectious Diseases, Korea National Institute of Health, Cheongju-si, Chungcheongbuk-do 28161 Republic of Korea; 4 Center for Emerging Virus Research, National Institute of Infectious Diseases, Korea National Institute of Health, Cheongju-si, Chungcheongbuk-do 28161 Republic of Korea; 5 College of Pharmacy, Research Institute of PharmaceuticalSciences, and Drug Information Research Institute, Sookmyung Women’s University, Seoul, 04310 Republic of Korea

**Keywords:** Nucleic acid therapeutics, Viral genome analysis, Conserved regions, Small interfering RNA (siRNA), Antisense oligonucleotides (ASOs), Off-target prediction

## Abstract

**Background:**

QStrain is an integrated resource platform designed to facilitate viral genome analysis and the rational design of nucleic acid therapeutics.

**Results:**

The system integrates reference-guided multiple sequence alignment of high-risk viral pathogens, including Severe Acute Respiratory Syndrome Coronavirus 2, Lassa virus, Severe Fever with Thrombocytopenia Syndrome virus, Middle East Respiratory Syndrome Coronavirus, Influenza A virus subtype H5N1 and H7N9, Nipah virus, Respiratory Syncytial Virus, and Dengue virus, with a growing database of viral genomes. QStrain provides versatile visualization modules such as frequency profiles, conservation scores, RNA expression patterns, and phylogenetic trees to enhance the interpretation of viral sequence variation. By identifying highly conserved genomic regions, QStrain supports the prediction of candidate therapeutic targets and enables the automated design of small interfering RNA (siRNA) and antisense oligonucleotides (ASOs). Additional features include off-target searching and 2D structure viewing for comprehensive assessment of therapeutic potential.

**Conclusions:**

Ultimately, QStrain (https://qstrain.sookmyung.ac.kr) provides a critical, user-friendly framework to accelerate the design pipeline of nucleic acid-based therapies, enhancing preparedness against current and future viral outbreaks.

**Supplementary Information:**

The online version contains supplementary material available at 10.1186/s12859-026-06424-0.

## Background

Emerging infectious diseases repeatedly disrupt global health and society. Epidemics such as severe acute respiratory syndrome (SARS, 2003)**,** influenza A/H1N1 (2009)**,** Ebola virus disease (2014)**,** Middle East respiratory syndrome (MERS, 2015)**,** Zika virus infection (2016)**,** and most recently coronavirus disease 2019 (COVID-19) have caused severe morbidity, mortality, and socioeconomic burden. These experiences underscore the urgent need for preparedness against future viral threats through both vaccines and effective antiviral therapeutics [1, 2]. Although advances in mRNA vaccines have accelerated immunization, therapeutic options capable of directly suppressing viral replication remain limited [3–5].

Nucleic acid therapeutics, including small interfering RNAs (siRNAs) and antisense oligonucleotides (ASOs), represent a promising strategy to fill this gap [6–10]. By binding sequence-specifically to viral RNA, they induce transcript degradation or translational repression. Their design principle as “informational drugs” enables rapid development: efficacy is defined by nucleotide complementarity, while pharmacokinetic behavior depends on the chemical scaffold [11, 12]. Once this scaffold is validated, new therapeutics can be generated by simply altering the sequence. The case of Milasen, a patient-tailored ASO developed within one year, demonstrates the feasibility of such accelerated timelines [11, 13].

A variety of bioinformatics tools and databases are currently available to support this process. Global repositories such as GISAID [14], NCBI virus [15] and ENA [16] provide viral genome data. Sequence alignment tools (Nextclade [17], ClustalW2 [18], MAFFT [19], and MUSCLE [20]) and phylogenetic platforms (Nextstrain [21], MEGA [22], and PhyML [23]) enable comparative analysis of viral evolution and indirectly guide the identification of conserved regions. Candidate siRNAs/ASOs can then be designed using PFRED [24], siRNADesigner [25], or ASOptimizer [26, 27], while RNAfold [28], RNAstructure [29], and Mfold [30] enable structural evaluation. Off-target potential can be assessed with BLAST [31], IntaRNA [32], and RNAhybrid [33]. However, these resources remain fragmented across multiple platforms, requiring researchers to manually combine diverse workflows, which is labor-intensive and prone to error.

To address these limitations, we developed QStrain, a unified web-based platform designed to facilitate viral genome analysis and therapeutic design. QStrain consolidates essential analytical modules—including reference-guided multiple sequence alignment (MSA), phylogenetic analysis, and specialized tools for siRNA/ASO design and off-target prediction—into a single framework. By integrating reference datasets for eight RNA viruses of major public health concern—Lassa virus (LASV), Severe Fever with Thrombocytopenia Syndrome Virus (SFTSV), Severe Acute Respiratory Syndrome Coronavirus 2 (SARS-CoV-2), Middle East Respiratory Syndrome Coronavirus (MERS-CoV), Influenza A virus (IAV), Nipah virus (NiV), Respiratory Syncytial Virus (RSV), and Dengue virus (DENV)—with these distinct but complementary tools, QStrain eliminates the need for fragmented workflows across multiple websites, thereby enhancing data accessibility and analysis efficiency.

As a systematic platform, QStrain enables rapid and rational design of nucleic acid therapeutics and enhances preparedness for future public health emergencies.

## Implementation

### Viral RNA genome dataset

We analyzed the genomes of eight RNA viruses (Supplementary Table 1). Complete genome sequences were downloaded in FASTA format from the NCBI Virus database (https://www.ncbi.nlm.nih.gov/labs/virus/); for LASV, partial genomes were also included due to limited availability.

After preprocessing (removal of duplicates, exclusion of incomplete or ambiguous sequences, and verification of genome type and length), curated sequences were defined as the reference genome dataset. According to the characteristics of each virus, sequences were classified by subtype, lineage, or genomic segment, yielding a total of 56 reference genome datasets.

### RNA expression dataset and expression profiling

RNA-seq datasets were collected from the Sequence Read Archive (SRA) and the Gene Expression Omnibus (GEO). Raw sequencing reads were retrieved using the SRA Toolkit [35] and quality-checked using FastQC, and preprocessed by trimming with Trimmomatic [36], adapter removal with Cutadapt [37], and additional filtering with the FASTX-Toolkit and Bowtie2 [38]. Clean reads were aligned to reference genomes using STAR [39], and alignments were processed with SAMtools [11] to generate read counts. Expression levels were normalized as log_10_(RPM + 1). The overall workflow and tool usage at each step are summarized in Supplementary Figure S1.

### Multiple sequence alignment

MSA was carried out using MAFFT v7.525 [19] with the FFT-NS-2 progressive algorithm, chosen as the default for the web service due to its speed and accuracy. For benchmarking, we also tested ClustalW2 [18] (full and quick modes, CPU and GPU) and MAFFT (L-INS-I for accuracy, FFT-NS-2 for speed). Runtime comparisons are reported in Results.

### Identification of conserved nucleotide regions

For each alignment position *i*, the nucleotide frequency was calculated as:

1 $${F_i}=\frac{\max\left({n}_{i,B}\right)}{N_i}$$

where B ∈ {A,G,C,T,gap}, *n*_*i,B*_ is the number of genomes with base B at position *i*, and *N*_*i*_​ is the total number of genomes with any base or gap at that position.

To assess conservation over therapeutic target lengths (16–22 nt), we calculated the homology score *H*_*s,k*_—a metric quantifying the degree of sequence conservation across the alignment—for a K-mer starting at s of length *k* as:

2 $${H}_{s,k}=\frac{\sqrt{{{\sum }_{i=s}^{s+k-1}({F}_{i})}^{2}}}{\sqrt{k}}$$

where *F*_*i*_ is from Eq. 1, *s* is the starting position of the *K*-mer, and *k* is the *K*-mer length. Both *Fi* and *H*_*s,k*_ range from 0–1, with 1 indicating complete conservation.

### Phylogenetic tree generation

Phylogenetic trees were reconstructed using FastTreeMP [40] with the maximum likelihood method. Bootstrap analysis (1,000 replicates) was performed to evaluate branch support, with values ≥ 0.7 considered strong.

### siRNA/ASO design

Candidate siRNAs and ASOs were designed using the PFRED algorithm [24], which incorporates sequence-derived parameters such as GC content, Tm, repetitive motifs, structural accessibility, and secondary structure propensity**.** Training data included 490 ASOs (15–21 nt) and 2,431 siRNAs (21 nt) with experimentally measured efficacies. Predictive models were built with support vector machines (SVM) and partial least squares (PLS).

To evaluate model performance, we used leave-one-out cross-validation. Predictive performance values were calculated as the proportion of variance in the observed activities explained by the model predictions, with higher values indicating stronger predictive accuracy.

### Off-target search

Potential off-targets were evaluated using a BLAST-based module against a locally deployed human transcriptome database (206,723 entries) [31]. Searches are executed with 20 threads, requiring 100% query coverage and applying a permissive E-value cutoff (1 × 10^12^) to retain all possible alignments.

### Secondary structure viewer

RNA secondary structures were predicted with the ViennaRNA package (RNAfold) [28], which applies minimum free energy (MFE) folding. Structural features such as hairpins, internal loops, bulges, and stem–loops were considered. Predicted secondary structures were visualized using the Forna library [41].

### Platform deployment

The platform was deployed on two servers: a web server (Intel® Core™ i7-7700 CPU, 32 GB RAM) for the user interface and data management, and a computation server (Intel® Xeon® Silver 4210 CPU, 64 GB RAM) for database construction and real-time analyses.

## Results

### Development of a web-based analysis platform

**Fig. 1:**
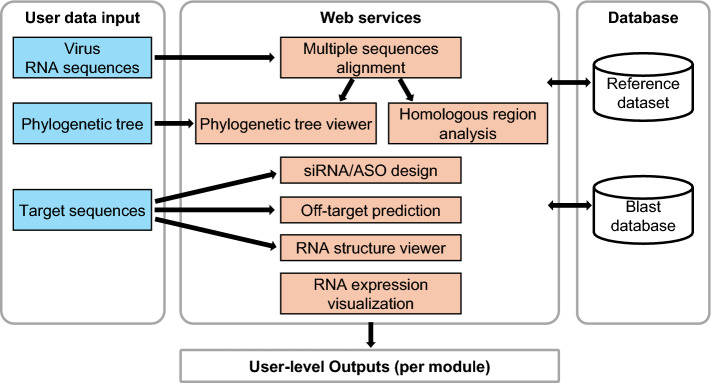
Overview of the web services. Most analyses can be performed with either user-provided input or reference datasets, while RNA expression profiles are available only from reference data

QStrain was implemented as a web-based platform that combines curated viral reference datasets with specialized analytical modules. It supports systematic exploration of genetic targets through functions such as multiple sequence alignment (MSA), homologous region analysis, phylogenetic tree visualization, siRNA/ASO design, off-target prediction via a local BLAST database, RNA secondary structure visualization, and RNA expression profiling (Fig. 1).

To support these functions, reference datasets were constructed for eight RNA viruses—LASV, SFTSV, SARS-CoV-2, MERS-CoV, IAV, NiV, RSV, and DENV. In total, 56 reference genome datasets were generated, with classification criteria tailored to each virus (Supplementary Table 1).

The platform enables both exploration of reference-derived results and incorporation of user-provided data for customized analyses. User sequences can be aligned with reference datasets to generate new MSAs or analyzed independently, which form the basis for phylogenetic tree construction and homologous region analysis. User-generated phylogenetic trees can be visualized using the integrated tree viewer. In addition, user-defined target sequences can be subjected to siRNA/ASO design, off-target prediction via the local BLAST database, and RNA secondary structure visualization. Together, these capabilities provide users with both flexibility and systematic support for therapeutic design.

### Analysis of nucleotide regions in viral genomes using MSA profile

**Fig. 2:**
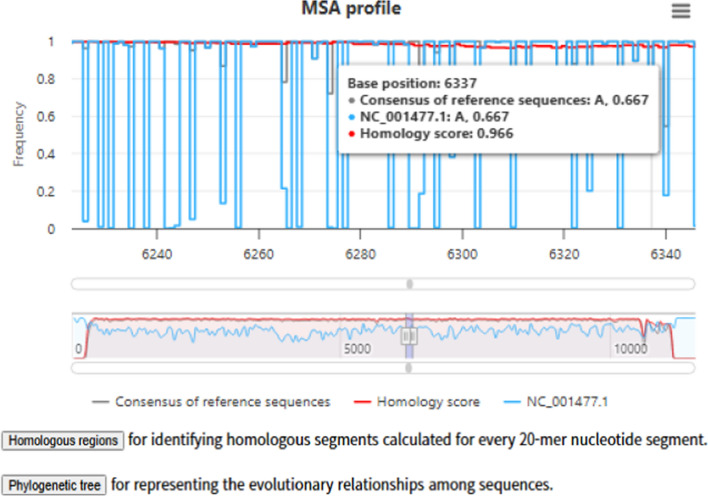
MSA profile of viral genomes. Example of DENV (Type2, 1,651 sequences) with a user-provided genome (NC_001477.1, blue). The grey line represents the consensus level based on nucleotide frequency, while the red line indicates the degree of sequence conservation (quantified as the homology score). High peaks in the red line correspond to highly conserved regions suitable for broad-spectrum therapeutic targeting

We implemented three modes of analysis for viral genome nucleotide regions using multiple sequence alignment (MSA): (i) reference-only, (ii) reference combined with user-provided sequences, and (iii) user-only datasets. The MSA profile provided three key metrics: a consensus score derived from reference sequences (indicating the most frequent base at each position), a homology score reflecting the degree of sequence conservation across aligned regions, and frequency values assigned to user-provided sequences (Fig. 2). This design enables both direct comparison with large-scale reference alignments and independent inspection of user datasets.

As shown in Fig. 2, using DENV Type 2, the consensus profile (grey) indicates the most common nucleotide at each position in the reference dataset, while the homology score (red) drops at positions with high variability. Superimposing a user-provided sequence (blue) instantly reveals its unique mutations or variations compared to the reference consensus.

Overlaying a user-provided sequence (blue) reveals deviations at single-nucleotide resolution, with the navigator bar allowing rapid inspection of conserved and variable regions. These functions collectively support fine-scale inspection and evolutionary interpretation of viral genomes.

### Alignment performance benchmarking

To assess computational efficiency, we compared ClustalW2 and MAFFT under different modes (Supplementary Table 2). Among all tested conditions, MAFFT quick mode on CPU was the fastest, completing the alignment of 165 full-length SARS-CoV-2 genomes in 0.11 h. In contrast, ClustalW2 in full mode with GPU required 1.5 h, despite being faster than its CPU counterpart. Thus, MAFFT quick CPU not only outperforms ClustalW2 but also provides the most practical solution for large-scale viral genome analysis, and was adopted as the default alignment engine for QStrain.

### Detection of conserved nucleotide regions across viral genomes

**Fig. 3:**
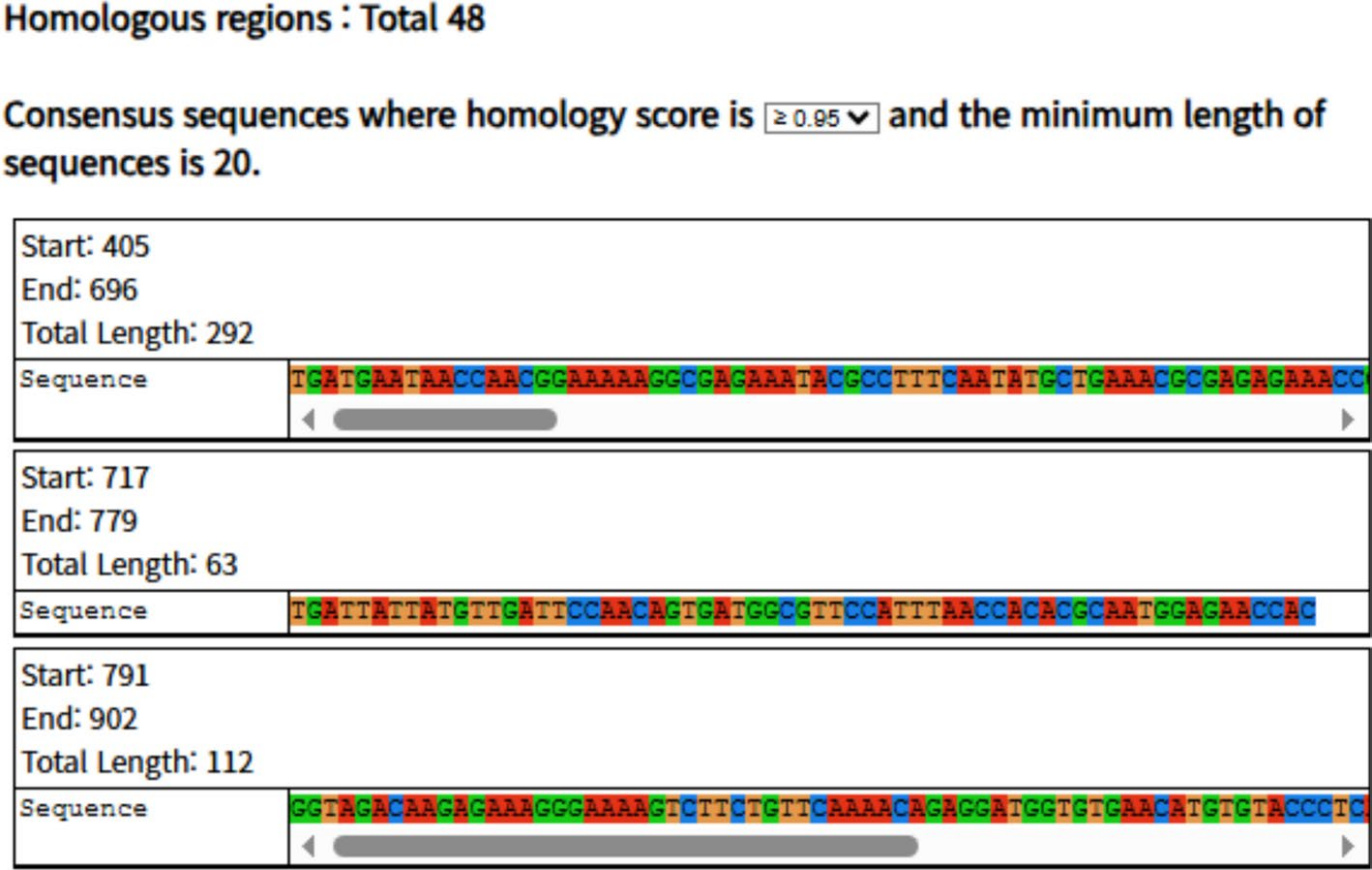
Identification of homologous nucleotide regions. Example of DENV (Type2, 1,651 sequences) with a user-provided genome (NC_001477.1). Shown are representative conserved regions (≥ 20 nt) identified as continuous nucleotide stretches where the homology score meets or exceeds the 0.95 threshold

Analysis of the MSA frequency profiles enabled the detection of conserved nucleotide regions across the viral genomes (Fig. 3). With a homology score cutoff of ≥ 0.95, we identified 48 conserved regions (≥ 20 nt). Relaxing the threshold to ≥ 0.9 decreased to 11 (not shown in the figure). Importantly, the number of regions does not always increase monotonically at lower cutoffs, since reduced stringency can merge adjacent regions into longer conserved blocks. As illustrated in Fig. 3 with DENV Type2, QStrain highlights conserved regions in lineage-specific datasets, allowing users to balance stringency and coverage depending on application needs.

### Phylogenetic tree analysis

**Fig. 4:**
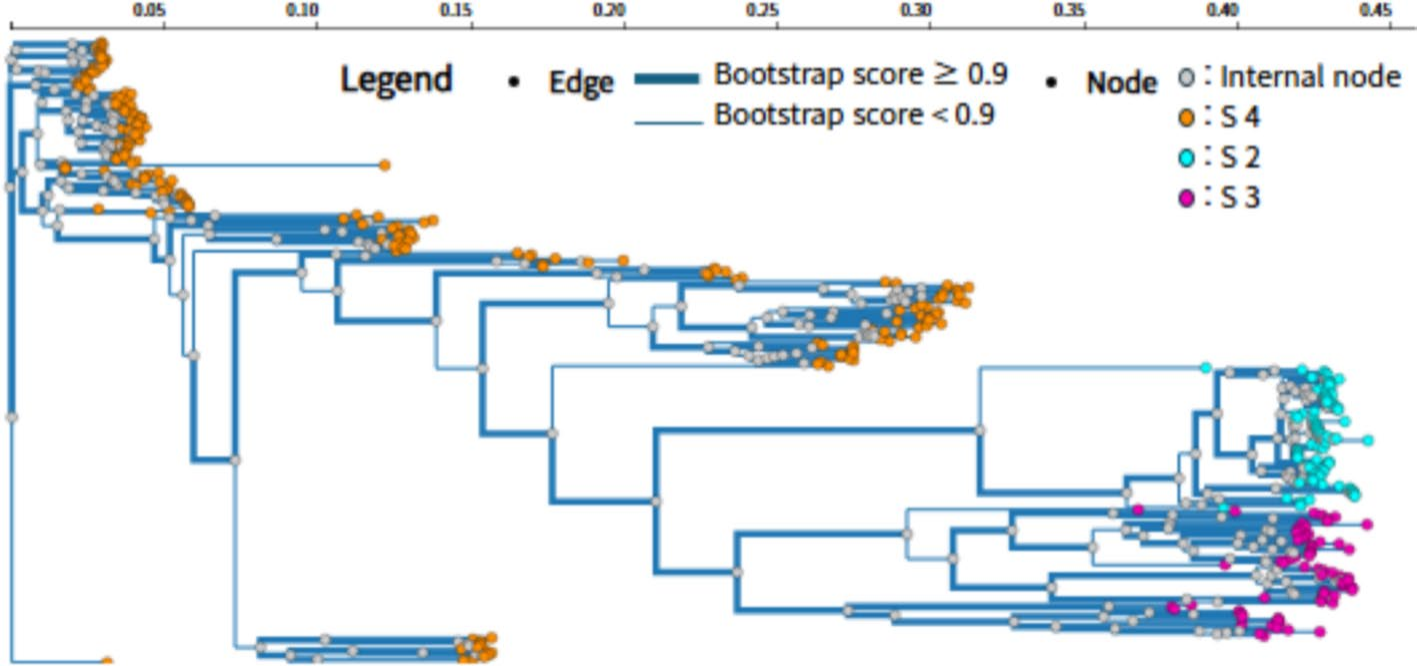
Phylogenetic tree analysis. Example from LASV segment S (clade 2, 3, 4; 325 sequences). Branches with a bootstrap score of ≥ 0.9 are highlighted in bold, indicating high confidence in the branching patterns. Terminal nodes are color-coded by clade subtype: Cyan dots represent Clade 2, Magenta dots represent Clade 3, and Orange dots represent Clade 4, while grey circles denote internal nodes

Phylogenetic tree reconstruction with FastTreeMP generated well-resolved topologies for the viral genome datasets (Fig. 4). Most internal branches were strongly supported (bootstrap ≥ 0.7), with several clades reaching ≥ 0.9. The trees clearly separated major viral subtypes and clades, consistent with established classifications. For example, LASV segment S formed distinct clades (clade 2, 3, and 4) comprising 325 sequences, illustrating QStrain’s ability to resolve lineage-level diversification. Closely related variants clustered tightly, whereas divergent lineages were positioned on longer branches, reflecting their evolutionary distance. Together, these results highlight that QStrain enables efficient and reliable phylogenetic inference with robust visualization of viral lineage diversification.

### RNA expression profiling of viral datasets

**Fig. 5:**
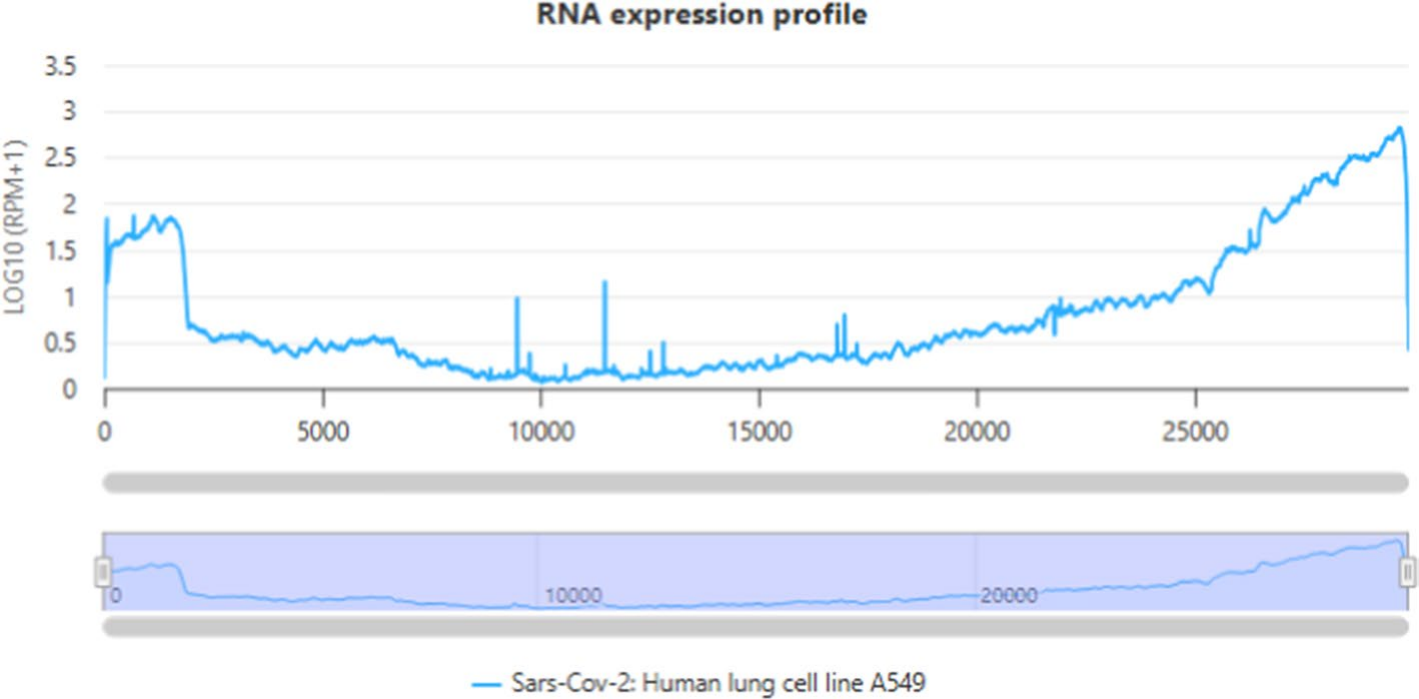
RNA expression profile of SARS-CoV-2 in A549 cells. RNA-seq data showing transcript abundance across viral genes, expressed as normalized read counts. Peaks indicating high transcript abundance serve as prioritized target regions for designing siRNAs or ASOs with maximal knockdown efficiency

To explore potential associations between genome sequences and transcriptional activity, we analyzed RNA expression profiles from 26 infection datasets (Supplementary Table 3). For segmented viruses (LASV, SFTSV, IAV), profiles were examined at the individual segment level. This revealed diverse patterns of transcript abundance across viral species and genome architectures. For example, RNA-seq data from SARS-CoV-2 infection in A549 cells (Fig. 5) highlight differences in transcript abundance across viral genes. These precomputed profiles enable users to explore viral transcriptional activity without raw data processing. Importantly, this profiling serves as a strategic guide for therapeutic design by identifying highly expressed viral genes. Targeting regions with high transcript abundance helps ensure accessibility and maximizes the knockdown efficiency of the designed siRNAs or ASOs.

### Design and evaluation of siRNA and ASO candidates

QStrain includes a dedicated module for siRNA/ASO design using the PFRED algorithm, which integrates thermodynamic and sequence-derived parameters. Using a 100-bp SARS-CoV-2 sequence (accession no. OU186099) as input, the module generated 81 candidate 20-mers (Supplementary Table 4). For each candidate, the module provides a predicted efficacy score (listed as ‘predicted activity’ by SVM/PLS in Supplementary Table 4), which estimates the probability of effective target silencing.

**Fig. 6:**
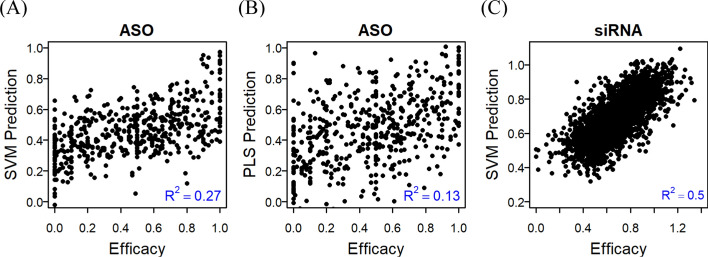
Leave-one-out cross-validation for PFRED models. **A** ASO efficacy with SVM, **B** ASO efficacy with PLS, **C** siRNA activity with SVM. Each dot represents the correlation between experimentally observed activity and the predicted efficacy score generated by the PFRED algorithm

To evaluate predictive models, we applied leave-one-out cross-validation (Fig. 6). For ASOs, the support vector machine (SVM) model achieved a predictive performance value of 0.27, outperforming partial least squares (0.13). For siRNAs, SVM achieved 0.50, indicating higher predictive accuracy. These results suggest that SVM-based PFRED models provide reliable estimates of predicted efficacy scores. However, it is important to note that these computational scores represent theoretical efficacy based on sequence and thermodynamic parameters; they do not guarantee biological activity in vivo, which must be verified through experimental validation.

### Off-target analysis of therapeutic candidates

QStrain implements a BLAST-based module to assess potential off-targets against the human transcriptome using a locally deployed BLAST database. This initial screening is crucial to minimize the risk of unintended gene silencing in human cells, a major safety concern in therapeutic development. Applying this to the same 100-bp SARS-CoV-2 input, we detected candidate sequences with full-length complementarity to human RNAs (Supplementary Table 5). The platform automatically highlights such hits, reporting sequence complementarity and allowing users to quickly exclude high-risk candidates. This ensures that only therapeutics with minimal predicted off-targets are prioritized for further evaluation.

### Secondary structure analysis of nucleic acid therapeutics candidates

**Fig. 7:**
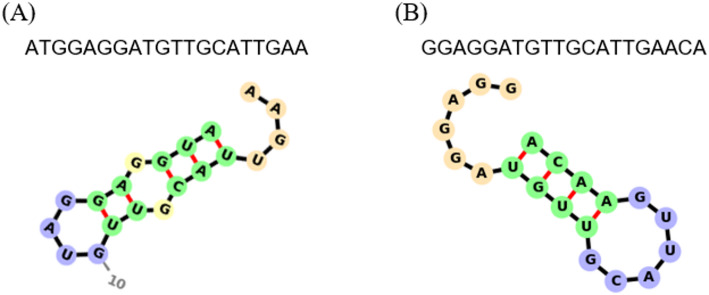
Representative RNA secondary structure predictions of 20-nt ASO candidate sequences using minimum free-energy folding. **A** and **B** show RNA structures of ASO candidates from homologous regions shared by RSV **A** and **B**

The platform provides an interactive oligonucleotide secondary structure viewer based on RNAfold predictions. When a sequence is entered, its predicted secondary structure is generated and displayed as an intuitive graphical representation (Fig. 7). This visualization highlights characteristic motifs such as stem–loop structures, enabling researchers to investigate structural features in siRNA guide strands or ASOs that influence effective binding to target RNA.

### Experimental validation of predicted candidates targeting Nipah virus

To further verify the biological relevance of QStrain’s predictions, we conducted in vitro validation targeting the Nipah virus (NiV). As shown in Supplementary Figure S2, candidates designed by QStrain demonstrated significant antiviral efficacy in A549 cells, with the top candidate (Nipah_11712) reducing target mRNA levels by over 80% compared to the control. This confirms that the platform effectively identifies biologically potent targets.

## Discussion

In this study, we present QStrain as a specialized resource platform that systematizes the rational design of nucleic acid therapeutics. While individual algorithms for alignment (e.g., MAFFT) and siRNA design (e.g., PFRED) are well-established, their scattered availability often hinders rapid response to viral outbreaks.

The primary added value of QStrain lies in its consolidated architecture. As shown in Fig. 1, the platform integrates genomic analysis and therapeutic design into a unified interface. In a conventional pipeline, researchers must manually retrieve sequences, align them using external software, and visually identify conserved regions. They then have to manually transfer these target sequences to separate design tools for evaluation—a process that is labor-intensive and prone to error.

QStrain addresses this by enabling a flexible workflow: users can first identify highly conserved "homologous regions" through the MSA module and then directly utilize these sequences in the dedicated siRNA/ASO design and off-target prediction modules. This centralization of analytical tools ensures that therapeutic candidates are designed based on thoroughly analyzed genomic data, reducing the complexity of manual data transfer.

Compared with existing resources that address these tasks separately, QStrain offers three major advantages. First, the platform links genomic, evolutionary, and expression data with therapeutic design modules, enabling systematic exploration of viral targets from sequence variation to candidate evaluation. Second, it allows both reference-based analyses and user-defined inputs, thereby combining reproducibility with flexibility. Third, its integration of off-target prediction and RNA structural accessibility facilitates early-stage screening of candidate siRNAs/ASOs, reducing the risk of advancing unsuitable designs.

Despite these strengths, several limitations remain. The current implementation is restricted to eight RNA viruses, and although these represent major pathogens of public health concern, broader coverage will be necessary to address diverse future threats. In addition, predictive models such as PFRED, while validated on existing datasets, still have limited accuracy compared to rapidly evolving AI-driven approaches [34] and require experimental confirmation. Furthermore, the current off-target analysis module relies on BLAST-based sequence complementarity and serves primarily as an initial safety screening tool to filter out perfect matches against the human transcriptome. While the design algorithms optimize the thermodynamic properties of the candidates themselves, the off-target screening does not yet account for partial matches, seed-region binding that characterizes miRNA-like off-target effects, or the thermodynamic stability of off-target interactions. Therefore, experimental validation remains essential to comprehensively assess the safety profile of any candidate prior to clinical application.

To ensure long-term utility and preparedness, QStrain is built upon a scalable modular architecture that allows for flexible data expansion. We maintain an update policy driven by public health relevance rather than fixed intervals; reference datasets will be updated upon the emergence of new variants of concern or significant accumulation of genomic sequences. Furthermore, the platform is designed to rapidly integrate new viral species as reference genomes become available, ensuring that QStrain remains a responsive tool for analyzing future viral threats.

## Conclusions

In summary, QStrain represents a significant step towards a ‘one-stop-shop’ solution for nucleic acid therapeutic design. By bridging the gap between genomic surveillance and rapid therapeutic development, it not only streamlines current research but also provides a scalable platform to bolster global preparedness for future pandemics.

## Availability and requirements

**Table:** 

Project name	QStrain
Project home page	https://qstrain.sookmyung.ac.kr/
Operating system	Platform independent
Programming language	HTML, PHP, JavaScript, Python
Other requirements	Not applicable
License	MIT License
Any restrictions to use by non-academics	No restrictions

## Supplementary Information

Additional file1

Additional file2

Additional file3

Additional file4

## Data Availability

The datasets supporting the conclusions of this article are available in public repositories as follows: NCBI Virus Datasets: All raw viral genome sequences were obtained from the NCBI Virus database (https://www.ncbi.nlm.nih.gov/labs/virus/). Detailed information on the 56 reference genome datasets, including specific classification criteria and accession numbers, is provided in Supplementary Table 1. RNA Expression Datasets: Raw sequencing reads for viral infection profiling were retrieved from the NCBI Sequence Read Archive (SRA) (https://www.ncbi.nlm.nih.gov/sra). The specific accession numbers for the 26 infection datasets analyzed in this study are listed in Supplementary Table 3. Software and Project Home Page: The QStrain platform is publicly accessible at https://qstrain.sookmyung.ac.kr/. Processed Data: Processed data generated from the multiple sequence alignment (MSA) and subsequent therapeutic design analyses are available from the corresponding authors upon reasonable request.
