# Supplementary material for: QStrain: an interactive platform for viral genome analysis and nucleic acid therapeutic design
Source: BMC Bioinformatics. 2026 Apr 10;27:105. doi: 10.1186/s12859-026-06424-0 (PMC13185203; doi:10.1186/s12859-026-06424-0)
Supplement: Supplementary file 1 — Additional file1 [file 12859_2026_6424_MOESM1_ESM.docx]

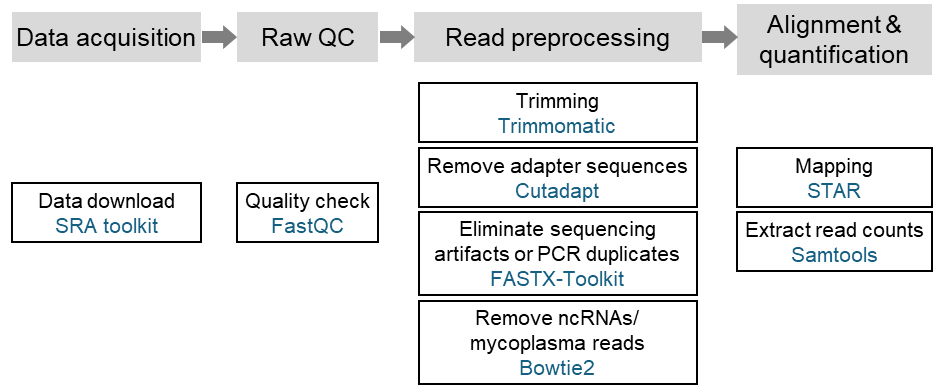


**Supplementary Figure S1. Workflow for RNA expression profile processing.**

**Supplementary Figure S2. Experimental validation of QStrain-designed candidates targeting Nipah virus.** Relative mRNA levels of the target gene in A549 cells treated with predicted candidates (10 μM, gymnotic delivery, 3 days). Target gene silencing was quantified using a dual-fluorescence reporter system (mGreenLantern/mCherry) and RT-qPCR. Data are normalized to mCherry expression and presented relative to the Non-Targeting Control (NTC). DW: Deionized Water (Mock). NTC: Non-Targeting Control. Dots represent individual data points from two replicates. Notably, candidate Nipah_11712 demonstrated >80% knockdown efficacy.
